# Following your heart as it takes shape

**DOI:** 10.7554/eLife.112337

**Published:** 2026-07-31

**Authors:** Alexandra Trouilloud, Nicole C Dubois

**Affiliations:** 1 Department of Stem Cell Biology and Regenerative Medicine, Department of Graduate Education, Icahn School of Medicine at Mount Sinai New York United States

**Keywords:** development, computational modeling, tissue patterns, live imaging, heart tube, congenital heart disease, Mouse

## Abstract

A novel computational pipeline reveals patterns of tissue movement and growth in early heart formation and advances virtual modeling of development.

**Related research article** Raiola M, Sendra MS, Domínguez JN, Torres M. 2026. Quantitative computerized analysis demonstrates strongly compartmentalized tissue deformation patterns underlying mammalian heart tube formation. *eLife* **14**:RP108559. doi: 10.7554/eLife.108559.

Have you ever eyed a wrapped gift, dying to know what was inside? Maybe, like a detective, you even gave it a shake to guess its hidden contents. Biologists regard the early mammalian embryo as a similar mystery box, devising increasingly clever tricks to peel back the wrapping and peek inside.

One of the early embryo’s most exciting secrets is the development of the heart. What starts as a simple sheet of cells will eventually evolve into the evolutionarily perfected four-chambered design that is pumping blood to keep you alive right now. The heart is the first organ to function in the embryo, so its proper formation is critical for supporting an organism throughout embryonic and postnatal life. To do so, it must accomplish a lot very quickly, generating large numbers of diverse cells and initiating coordinated beating, all while establishing its complex shape. There are plenty of opportunities for this process to go awry, resulting in a high rate of congenital heart defects – many with unclear origins.

Studying the earliest steps of organ formation at cellular detail within live mammalian embryos has posed a major challenge. Previous approaches to study the early embryo include anatomically characterizing dissected hearts, tracing cell populations with genetic or dye-based labels, sequencing and perturbing genes, and live-imaging embryonic hearts in model organisms. Together, these efforts have led to a deep understanding of the genetic networks and cell lineages governing heart development (Bruneau et al., 2001; Cai et al., 2003; Meilhac et al., 2003; Asp et al., 2019) as well as descriptions and early models of heart morphogenesis (de Boer et al., 2012; Ivanovitch et al., 2017; Le Garrec et al., 2017). Still missing, however, is a quantitative and continuous picture of the dynamic processes of early heart morphogenesis, including how cells and tissues grow, move, and collectively shape the developing heart.

Now, in eLife, Miguel Torres and colleagues at the Centro Nacional de Investigaciones Cardiovasculares in Spain – including Morena Raiola as first author – report new insights into heart formation using an original computational pipeline to track and identify patterns of tissue deformation in live images of developing mouse embryos (Raiola et al., 2026). Raiola et al. built a model that predicts tissue rearrangements at cellular resolution, beyond the depth previously attainable in early heart development.

The new analysis pipeline overcomes a common limitation of live imaging by stitching together multiple datasets into a continuous model of heart development (Raiola et al., 2025). It combines original time-lapse images of mouse embryos collected over 12 hours of heart tube formation with a previously published anatomical atlas spanning the same window (Esteban et al., 2022). This framework allowed Raiola et al. to quantify tissue growth and deformation over time as the cardiac crescent – an early structure – begins to transform into the heart tube.

Applying this model to their present study, Raiola et al. uncovered previously unrecognized patterns of tissue rearrangement, revealing a striking compartmentalization of collective cell behavior. Growth is concentrated on either side of the heart tube, while deformation dominates the central region, stretching the middle of the heart tube along the head-to-tail axis. These coordinated movements cause the medial heart tube to bulge outward, while two ring-like regions on the sides remain relatively constrained, giving the developing heart tube the shape of a barrel.

To understand how these tissue-level movements emerge from individual cell behavior, Raiola et al. developed their most powerful computational tool yet – a first-of-its-kind fate map that tracks individual cell trajectories within deforming tissue. This model predicts movements of simulated cells within the dynamic heart tube model, closely matching measured trajectories from labeled cells in living embryos. Using this tool, researchers can add extraordinary cell-level detail to overall tissue deformation patterns. Accordingly, the researchers followed several pseudo-cell clusters along the heart tube and observed cellular rearrangements that support their proposed barrel model of heart tube formation.

Raiola et al. provide a new imaging-based framework for a dynamic developmental model that can describe multiscale cell and tissue movement and predict these patterns in a computational model (Figure 1). Beyond revealing patterns in heart tube formation, this approach opens the door for future predictive models to better understand how congenital heart defects arise from these critical early steps. Applied across tissues and organs, it could establish a powerful platform for future studies into the early mysteries of development.

**Figure 1.**
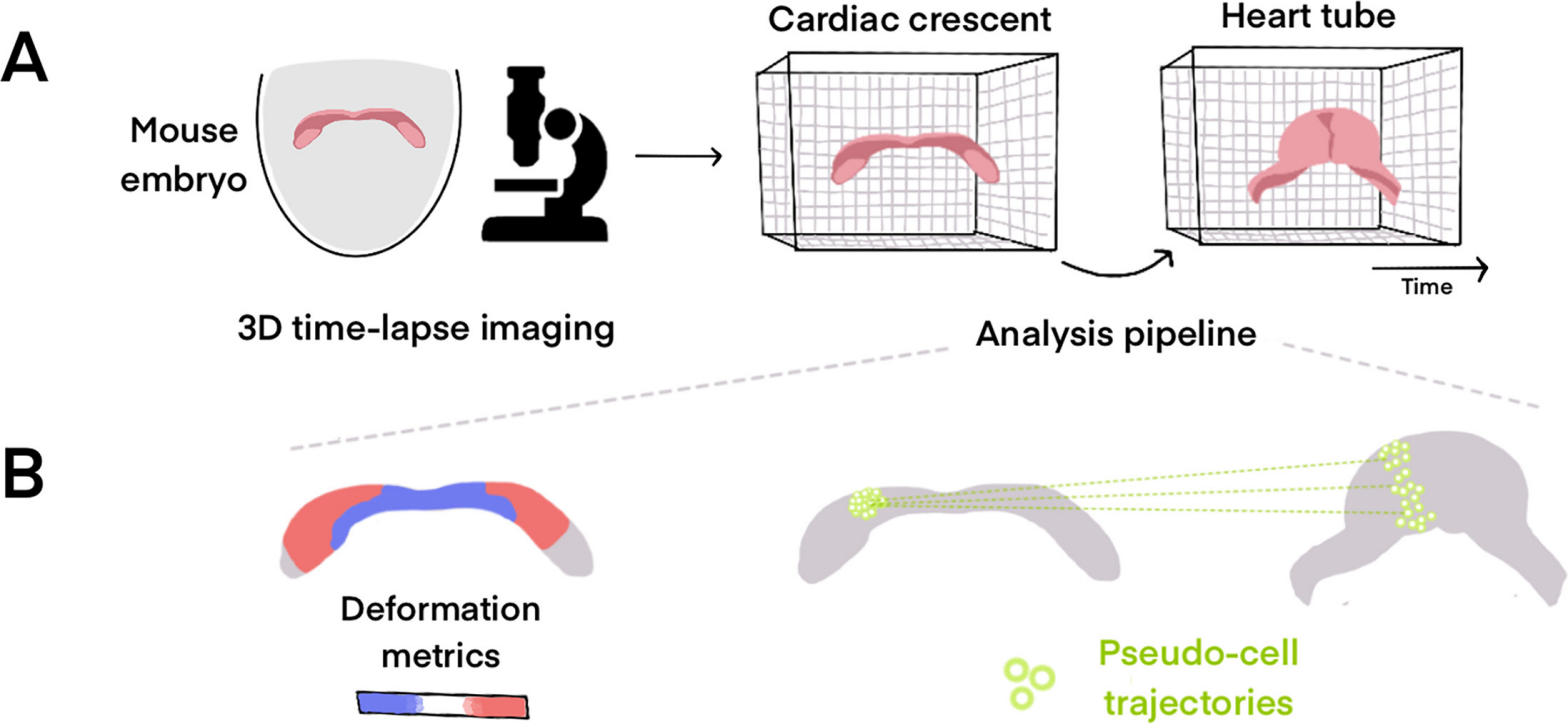
Analysis and modeling of early heart development in the mouse. (**A**) Mouse embryos are imaged during a 12 hour window of development from the cardiac crescent to the early heart tube stage. A novel computational pipeline extracts stage-by-stage and overall patterns of tissue deformation between the 3D images over time. (**B**) Specific metrics of tissue deformation, such as growth and direction, are measured and used to reconstruct a developmental model. A virtual fate map based on this model predicts how “pseudo-cells” charted onto the heart move throughout development.
